# A Correlation of Overall Mass Transfer Coefficient of Water Transport in a Hollow-Fiber Membrane Module via an Artificial Neural Network Approach

**DOI:** 10.3390/membranes13010008

**Published:** 2022-12-21

**Authors:** Xuan Linh Nguyen, Ngoc Van Trinh, Younghyeon Kim, Sangseok Yu

**Affiliations:** 1 Department of Mechanical Engineering, Graduate School, Chungnam National University, Daejeon 34134, Republic of Korea; 2 School of Mechanical Engineering, Chungnam National University, Daejeon 34134, Republic of Korea

**Keywords:** hollow-fiber membrane, mass transfer, artificial neural network, humidifier, vehicular fuel cell

## Abstract

Water transport in a hollow-fiber membrane depends on mass convection around the tube, mass convection inside the tube, and water diffusion through the membrane tube. The performance of water transport is then explained by the overall mass transfer coefficient in hollow-fiber membranes. This study presents the prediction of overall mass transfer coefficients of water transport in a hollow-fiber membrane module by an artificial neural network (ANN) that is used for a humidifier of a vehicular fuel cell system. The input variables of ANN are collected from water transport experiments of the hollow-fiber membrane module that is composed of inlet flow rates, inlet relative humidity, system pressures, and operating temperatures. The experimental mass transfer coefficients are the targets of the training model, which are determined via the effectiveness analysis. When unknown data are applied to the ANN model, the correlation of the overall mass transfer coefficient predicts precise results with R = 0.99 (correlation coefficient). The ANN model shows good prediction capability of water transport in membrane humidifiers.

## 1. Introduction

Hollow-fiber membrane modules have been extensively used for vehicular humidifiers to provide a large surface area within a small volume [1,2,3]. Membrane-based configuration can be scaled up more easily than traditional mass transfer equipment [4]. The membrane shell-and-tube heat mass exchangers are introduced in humidification techniques to enhance the heat and moisture exchange effectiveness. The heat and mass transfer in the hollow-fiber membrane module is crucial in performance analysis and system optimization [5].

Many researchers have published their heat and mass transfer research by showing the sensitivity investigation of the effects of parametric and geometric conditions or proposing Nusselt and Sherwood empirical correlations, representing an exchange of both sensible and latent heat in the fluid flow. Cahalan et al. [6] conducted an experimental evaluation of a humidifier at various Reynolds numbers and evaluated its humidification performance based on their proposed lumped model. Chen [7] presented the effect of channel structure on the performance of a membrane humidifier via heat and water flux, water recovery ratio, and pressure loss. Vu et al. [8] proposed an empirical correlation of the overall mass transfer coefficient to predict the performance of a hollow-fiber membrane module used for a vehicular fuel cell system. This coefficient is sensitive to the input parameters such as temperature, pressure, relative humidity, and air flow rate. Kui He [9] conducted a topological study with different packing densities and inlet flow Reynolds numbers from 0.35 to 0.5 and 20 to 180, respectively. The fluid flow and heat transfer were predicted to optimize hollow-fiber membrane contactors with randomly distributed fibers.

Even though experimental investigations are essential to adapt to actual systems, conducting experiments under all required conditions is time-consuming and not economically viable. Hence, early prediction of the system performance can be accomplished by using powerful computing techniques such as artificial neural networks to employ a limited number of experimental results.

Artificial neural networks (ANNs) are systems of weight vectors whose component values are determined by different machine learning algorithms. These networks take a linear set of pattern inputs and output a numerical pattern representing the actual output [10]. The ANNs resemble how the human brain learns. It can learn basic patterns within a multi-information domain rather than intricate rules and mathematical formulas. ANNs have been widely chosen because of their aptitude for simulating non-linear systems and ease of application to real-world scenarios. This method is known to be a powerful tool to optimize the operation of proton exchange membrane fuel cells [11,12,13]. Sisworahardjo et al. [11] showed a multilayer feedforward ANN with a backpropagation training algorithm trained for 10,000 epochs using their experimental data. This model was used for the dynamic model of the portable PEMFC. Nanadegani et al. [12] applied the ANN to find the operating conditions which maximize the power output for various currents. The critical conditions were operating temperature, relative humidity, and stoichiometry at the cathode and anode side at a constant pressure of 1 bar. The optimization could be used to reduce the number of cells for a given power output. Han et al. [13] proposed a model based on a neural network and semi-empirical equation to optimize the system efficiency including the stack, humidifier, blower, power converter, pump, and other balance of plants. They claimed that the model can be extended to similar PEM fuel cell systems even though it was based on empirical modeling for a specific system. These models focused more on the fuel cell stacks but not a component in the systems. A membrane humidifier is one of the critical components that must be optimized to reach high heat and mass exchangeability. Therefore, the heat and mass transfer performance should be predicted well to propose a precise design strategy. However, the ANN approach was rarely applied to a membrane humidifier. Several researchers predicted heat and mass transfer using artificial neural networks in various systems. Liu et al. [14] developed a model of ANN to predict the mass transfer coefficient of the ozone absorption process. The inputs included dimensionless parameters such as Reynolds, Weber, and Froude numbers to reflect the liquid- and gas-phase characteristics. They optimized the network with three hidden layers and 15, 25, and 25 neurons in each layer to predict the performance with R squared of 0.9896 and 0.9877 on the training test and test set, respectively. Meesattham et al. [15] used the Levenberg–Marquardt algorithm to train the data with one hidden layer in a model of predicting the mass transfer coefficient of CO_2_ absorption into aqueous solutions. This model showed an outstanding performance over the predictive proposed correlations in the literature. ElShazly [16] estimated the mass transfer coefficient from the bottom of the agitated vessel and compared it with the empirical correlation expressed by Sherwood, Schmidt, and Reynolds numbers from a dimensional analysis. The author designed a network with one hidden layer and with varying neurons from 1 to 12 to optimize the structure. The ANN reached the best performance when applying three to seven neurons in the hidden layer. The model results were better than the prediction using mass transfer correlation because they showed smaller relative errors. 

In this study, a prediction model of overall mass transfer coefficients in a hollow-fiber membrane module was developed with an artificial neural network (ANN) approach. This technique is effective because water transport in the membrane humidifier is a non-linear problem. The overall mass transfer coefficient was investigated experimentally, and then an ANN model was developed to train the experimental data. The experimental conditions were designed with five levels in the range of four parameters, including temperature (40–80 °C), pressure (100–200 kPa), flow rate (5–15 slpm), and relative humidity (0.6 to 1). Therefore, there were 5^4^ cases of tests that should be carried out for developing an empirical correlation. About 3 to 3.5 h were necessary to conduct a case of experiments, so the entire experiment completion would need about 2000 h. However, the deviation in the previous study [8,17] was still high in fitting the water vapor diffusion with R^2^ from 0.85 to 0.9. Thus, the ANN model could be used to predict the mass transfer performance of the module after collecting the data from about a hundred experimental cases. This model provided a correlation based on the weights and biases of the optimized neural network with a very high coefficient of correlation (about 0.99) and low mean squared error.

## 2. Measurement and Calculation of the Overall Mass Transfer Coefficient

### 2.1. Effectiveness Analysis for Mass Transfer Coefficient Determination

The effectiveness–*NTU* (*ε-NTU*) correlation could be used for a standard heat exchanger to predict performance and design effectively. This study analyzed the mass exchange utilizing the heat and mass transfer analogy. The *ε-NTU* relation for counter flow can be defined as:

(1) $$NTU=\frac{1}{C-1}ln\left(\frac{\varepsilon -1}{\varepsilon C-1}\right)$$

where *C* is the mass capacity ratio and ε is moisture effectiveness.

The mass capacity ratio is the proportion between the mass flow rates:

(2) $$C=\frac{{\dot{m}}_{min}}{{\dot{m}}_{max}}$$

The moisture effectiveness of the module was calculated from experimental data:

(3) $$\varepsilon =\frac{{\dot{m}}_{d}\left(\omega_{dry,\;out}-\omega_{dry,in}\right)}{{\dot{m}}_{min}\left(\omega_{wet,in}-\omega_{dry,in}\right)}$$

where ${\dot{m}}_{d}$ is the mass flow rate of dry air and ω is the absolute humidity.

The absolute humidity of water vapor can be expressed by:

(4) $$\omega =0.622\frac{\varphi p_{s}}{p_{t}-\varphi p_{s}}$$

where φ is relative humidity, $p_{t}$ is total pressure, and $p_{s}$ is saturation pressure.

The saturation pressure is a function of temperature from the Antoine equation:

(5) $$p_{s}=10^{7.16728-\frac{1716.984}{232.538+T}}$$

Then, the overall mass transfer coefficient of the membrane module was calculated based on the relation with *NTU*:

(6) $$NTU=\frac{\rho_{a}k_{o}A_{m}}{{\dot{m}}_{min}}$$

where $\rho_{a}$ is the dry air density, $k_{o}$ is the overall mass transfer coefficient, and $A_{m}$ is membrane area.

The mass transfer rate through the membrane module was calculated using effectiveness and the overall mass transfer coefficient was estimated from the ANN model:

(7) $$\dot{m_{t}}=\varepsilon {\dot{\;m}}_{min}\left(\omega_{wi}-\omega_{di}\right)={\dot{\;m}}_{min}\frac{1-exp\left[NTU\left(C-1\right)\right]}{1-Cexp\left[NTU\left(C-1\right)\right]}\left(\omega_{wi}-\omega_{di}\right)$$

### 2.2. Experiment Description

**Figure 1:**
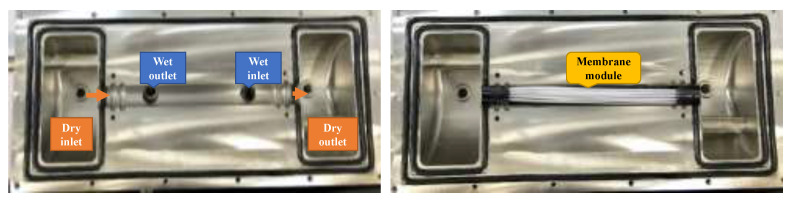
A configuration of a hollow-fiber membrane humidifier.

In this study, a small-scale hollow-fiber membrane humidifier was designed to test the water vapor transfer from the wet-air side to the dry-air side through the membrane. The membrane tubes made of polytetrafluoroethylene (PTFE) polymer were chosen with 1.1 mm in outer diameter, 0.1 mm in thickness, and 110 mm in length. Twenty-one hollow-fiber membranes were compacted evenly to form a module inside a specially designed jig, which is a shell–tube configuration of our membrane humidifier. Dry air was set up to flow inside the membrane tube (tube side) while wet air entered outside the membrane (shell side). The humidifier setup is shown in Figure 1.

**Figure 2:**
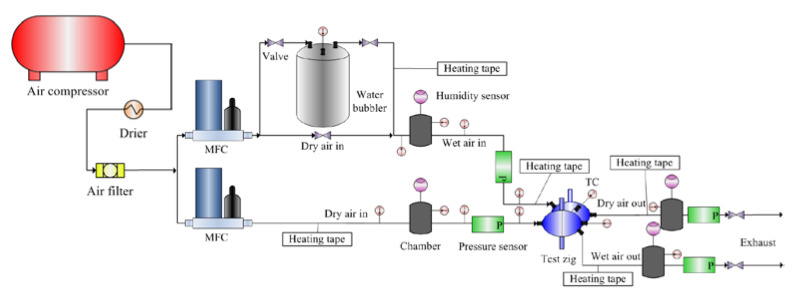
A diagram of the experimental system.

As in Figure 2, a schematic of the experimental structure was used to measure the mass transfer performance of the membrane module. Ambient air was driven through a compressor and a drier to remove moisture from the flow. The air passed through a filter before being divided into two channels for the dry and wet sides of the membrane module. These air flows were heated to operate at desired temperatures. Extra vapor was added to the wet side by a water bubble humidifier to create the concentration gradient between the two sides of the membrane. This humidifier and a bypass valve controlled the inlet humidity of wet air to adapt to the conditional input range. The two flows were then injected into the membrane module using mass flow controllers to investigate the moisture exchange. Four chambers were used to keep flows stable and measure the inlet and outlet air properties. The isothermal condition was maintained throughout the experiments with enwrapped heating tapes. The NI PXI system driven by LabVIEW software was employed to collect the data of temperature, relative humidity, pressure, and mass flow rate from installed Vaisala sensors, P126 pressure transmitters, and mass flow controllers, respectively. The overall mass transfer coefficient was analyzed with the above data reduction process.

## 3. An ANN for Prediction of the Overall Mass Transfer Coefficient

### 3.1. Structure of Artificial Neural Network

**Figure 3:**
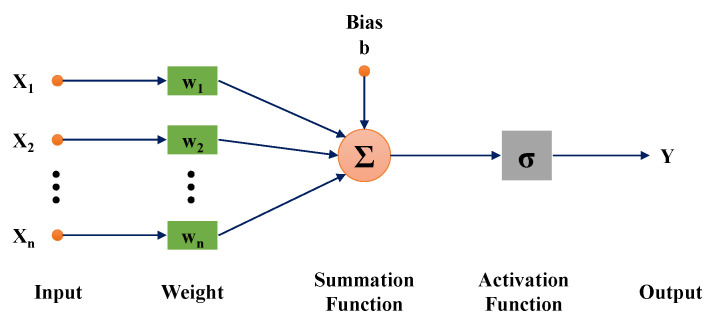
Basic structure of the ANN model.

The ANN architecture consists of layers, including input, hidden, and output layers. These layers contain neurons that serve various functions. The connections between the layers and the neurons are affected by the weights and biases. The input neurons represent independent variables. The hidden neurons are created by combining inputs, weights, biases, and transfer functions. The output neuron is the target, or independent variable. Figure 3 shows a basic algorithm for the ANN model. Each of the inputs is multiplied by a weight that can be adjusted once a model is trained. These products are summed with a bias and sent to an activation function so that the result is then transformed into an output.

(8) $$Y\left(X\right)=\sigma \left(\overset{n}{\underset{j=1}{\sum }}\left(X_{j}w_{j}+b\right)\right)$$

where *X*, *Y* are independent and dependent variables, *b* is bias, w is weight, and σ is activation function.

The ANN modeling process consists of two steps: training the network in the first step and then testing it with data that were not used for training. A training set is a group of matched input and output patterns used for training a network. When each pattern is read, the network uses the input data to produce an output, which is then compared to the training pattern, i.e., the correct or desired result [18]. A neural network can be trained by changing the values of the connections between the elements to minimize the deviation between the calculated output from the network and the experimental result. When the training reaches an accuracy criterion, the network retains those weights, and the trained network can be used to make decisions. 

In this study, the data were required to be pre-processed after being collected from the experiments. The normalized data were then trained by several neural network models to determine the best one. Cross-validation with experimental data was the next step to ensure the reliability of the model for further analysis.

### 3.2. Data Collection and Pre-Processing

The data used to train the neural network were collected from experiments of water transfer in the membrane humidifier. The inputs are operating parameters, including temperature, pressure, relative humidity, and flow rate, because each parameter plays a vital role in humidification performance. The output is the overall mass transfer coefficients determined via the ε-NTU analysis. The range of inputs and output are detailed in Table 1. 

The total available data set from experiments were randomly divided into three independent subsets as training data, validation data, and test data in the ratio of 70:15:15 respectively. All values were normalized between 0 and 1 for training and prediction of the ANN by the following formula:

(9) $$X_{normalized}=\frac{X_{j}-X_{min}}{X_{max}-X_{min}}$$

### 3.3. A Neural Network for Training Experimental Data

In this study, a neural network was constructed using the MATLAB Neural Network Toolbox (R2019a). The network was applied with the Levenberg–Marquardt backpropagation algorithm to train the overall mass transfer coefficient and then predict the mass transfer rate through the membrane module. Valera et al. [19] and Hagan et al. [20] found that Levenberg–Marquardt is the most common algorithm because it showed much more efficiency than others when the network contained no more than a few hundred weights. The activation function in the hidden layer was tangent sigmoid, given by the equation

(10) $$\sigma \left(z\right)=\frac{1}{1+e^{-z}}$$

where *z* is the weighted sum of the input.

The ANN model should be optimized so that it can predict the mass transfer well even using the minimum number of parameters and simple structure. It contributes to the training time and cost reduction. In this study, the optimum number of training layers and neurons was chosen after a sensitivity analysis in which the performance of the model with one or two layers and 5 to 10 neurons per layer was evaluated. Table 2 summarizes the results of the sensitivity analysis with four network models in terms of the linear correlation coefficient (*R*) and the mean squared error (*MSE*) between model data and neural network outputs. The *R* and *MSE* values are defined below [10,12].

(11) $$R={\left(1-\frac{\sum {\left(t_{j}-o_{j}\right)}^{2}}{\sum {\left(o_{j}-\bar{o}\right)}^{2}}\right)}^{1/2}$$

(12) $$MSE=\frac{1}{n}\overset{n}{\underset{j=1}{\sum }}{\left(desired\;output-network\;output\right)}^{2}$$

From Table 2, it can be seen that more hidden layers or the number of neurons did not always enhance the results because the coefficient of correlation was lower, and the mean squared error was higher with increasing the two parameters. Although Network 4 with three hidden layers showed a better performance than Network 1, with a slight increase in *R* and reduction in *MSE* values, duplicating the epoch was necessary for convergence. It would cause complications in ANN structure and take much time in the case of training thousands of data sets. 

**Figure 4:**
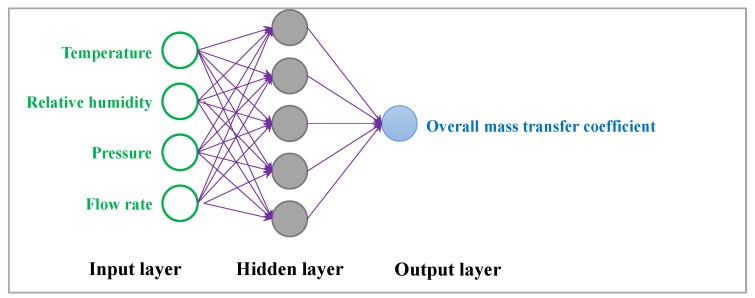
The optimized neural network for training the experimental data.

Using a single training layer with only five neurons can reduce the size of the weight and biases matrix. The small number of neurons and layers could also allow for easier extraction of the final equation in the neural network. The optimal Network 1 was chosen with the parameters shown in Table 3. The training network consisted of three layers: an input layer with four neurons, a hidden layer with five neurons, and an output layer with one neuron, as shown in Figure 4.

**Figure 5:**
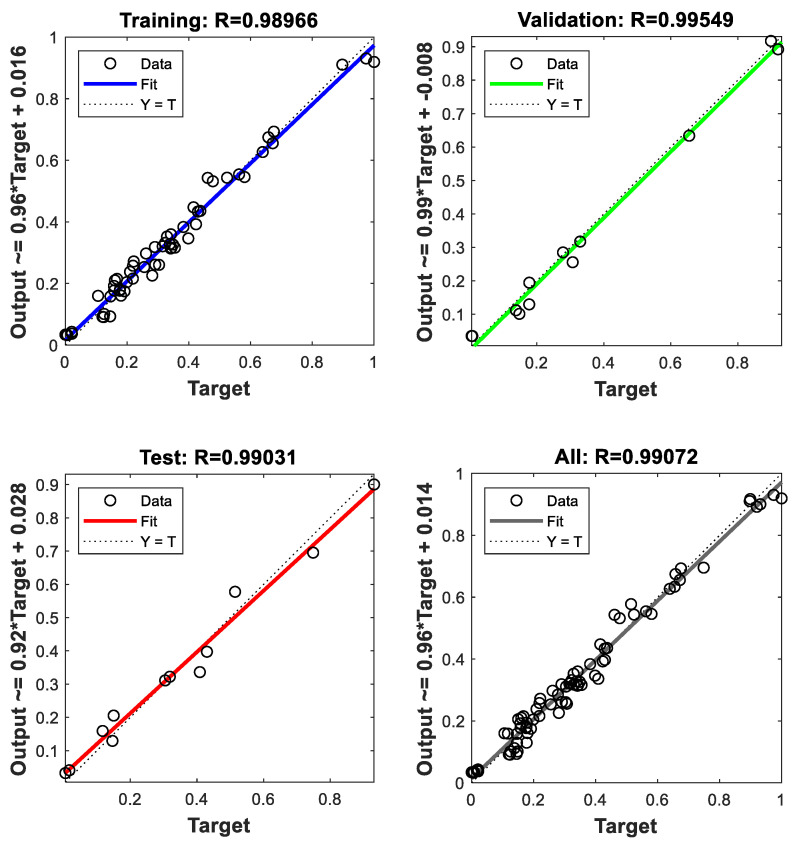
Goodness of fit of the optimized training model.

As the neural network started to train, a weight and a bias matrix were initialized randomly to produce the mass transfer coefficient from the conditional inputs. They were compared with the experimental mass transfer coefficient to adjust the initialized matrixes and find the optimized correlation for further prediction. Figure 5 presents the relationship of the training, validation, testing and all values of the ANN models for observed and predicted mass transfer coefficient. It can be seen that the R value of training, validation, testing, and all are about 0.99. The results indicate that the correlation between observed and predicted values is strong in all data sets.

### 3.4. Validation of the Trained Model with Experimental Analysis

**Figure 6:**
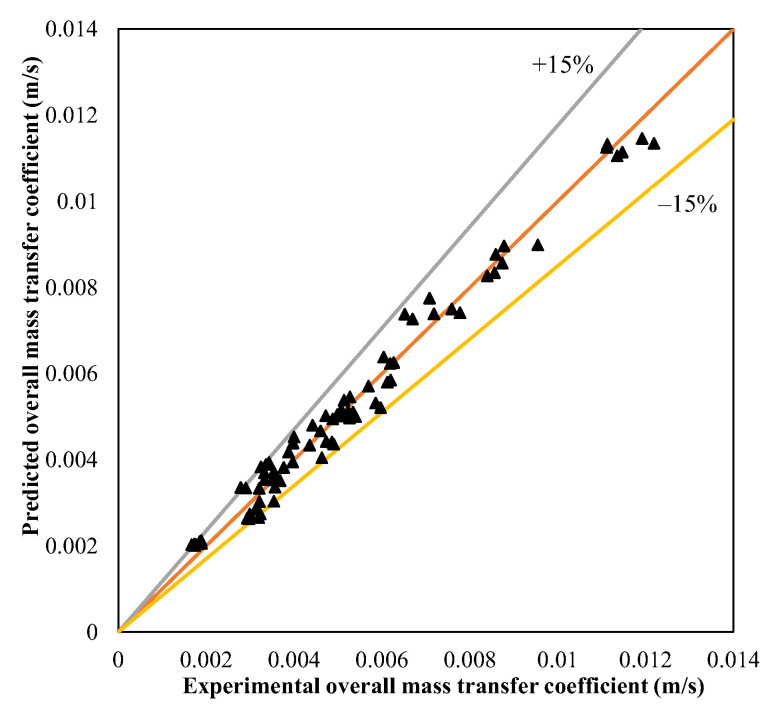
Validation of predicted mass transfer coefficient with experimental data.

Figure 6 shows the actual response and predicted responses with the average error for all test data using the trainlm network with the optimum number of neurons. It can be seen that the trained network can well predict the overall mass transfer coefficient within the average error of ±15%. As a result, neural networks with an optimal number of neurons and an appropriate design can be used to forecast the performance parameters of mass transfer with a minimum number of experiments, which is also advantageous in budgetary considerations.

## 4. Mass Transfer Performance Prediction of the Membrane Module

**Figure 7:**
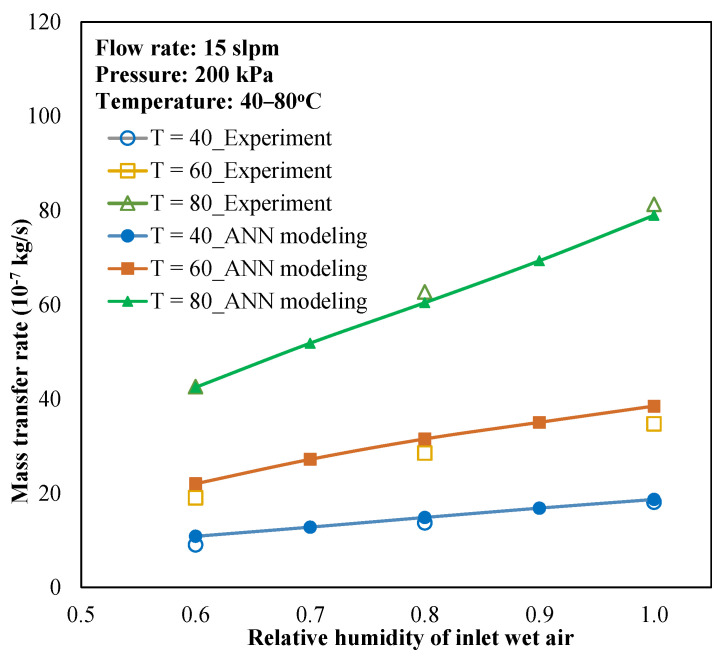
Validation of modeling and experiments in water transport rate.

The trained ANN was applied to predict the overall mass transfer coefficient with various conditional parameters, and then the water transfer rate through the membrane was determined using the ɛ-NTU relation. Figure 7 presents the prediction of the water transfer rate through the membrane module in comparison to experiments. From the ANN-predicted mass transfer coefficient, the water transfer rate can also be determined for the same membrane module with a small deviation. 

**Figure 8:**
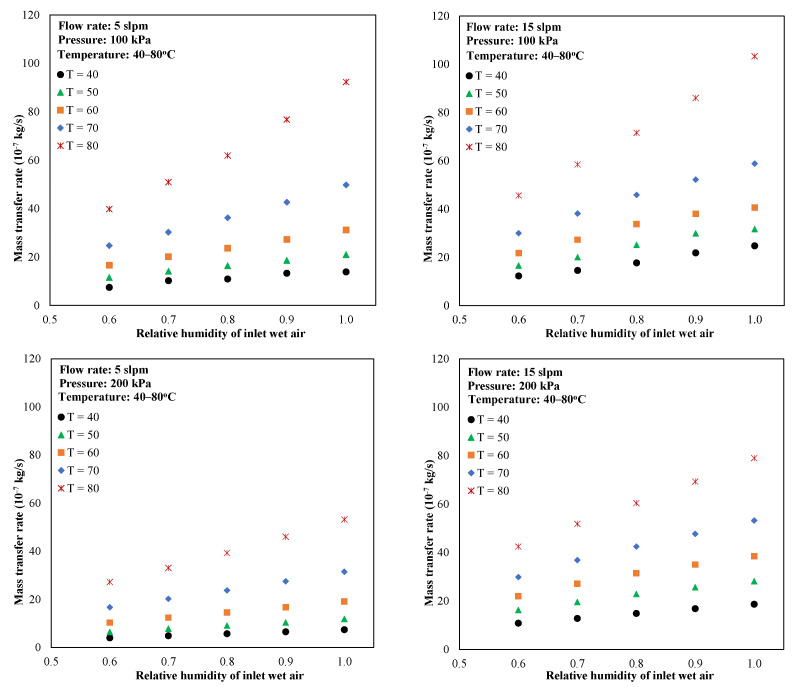
ANN-based prediction of water transport through the membrane.

Figure 8 shows the membrane module performance versus relative humidity with a variation in temperature, pressure, and flow rate. It can be seen that the water transfer rate increased with an increase in temperature, flow rate, relative humidity, and a decrease in pressure. This tendency was confirmed in the literature [17,21], meaning that this correlation model can be used to predict the transport performance of various hollow-fiber membrane humidifier designs and operating conditions with high accuracy.

## 5. Conclusions

The ANN was successfully applied to predict the overall mass transfer coefficient of vapor transport through the membrane module using mathematical formulations from machine learning algorithms. These have been obtained from formulations of the selected functions, such as summation and activation, in the ANN model and weights of the neurons. The predicted mass transfer coefficient was found to be in good agreement with the experimental data. The trained neural network was also used to predict the mass transfer performance of the membrane module in the designed humidifier.

## Figures and Tables

**Table 1:** Range of variables for the neural network.

Parameters	From	To	Unit
Temperature	40	80	°C
Absolute pressure	100	200	kPa
Flow rate	5	15	slpm
Relative humidity	0.6	1	
Overall mass transfer coefficient	0.00167	0.0122	m/s

**Table 2:** Optimizing the number of training layers and neurons.

Model	No. of Layers	No. of Neurons	*R*	*MSE*	Epoch
Network 1 *	1	5	0.99072	9.58 × 10^−4^	10
Network 2	2	5	0.87269	8.32 × 10^−3^	2
Network 3	2	10	0.97295	4.19 × 10^−3^	7
Network 4	3	5	0.99632	7.42 × 10^−4^	20

* Optimum.

**Table 3:** The network model parameters (Network 1).

Parameter	Parameters
Training function	trainlm
Transferring function	tangent sigmoid function
No. of layers	1
No. of neurons	5

## Data Availability

Not applicable.

## Nomenclature and Abbreviation

*ANN*	artificial neural network
*A*	area, m^2^
*b*	bias
*C*	mass transfer capacity ratio
*k*	mass transfer coefficient
*ṁ*	mass flow rate, kg/s
*MSE*	mean squared error
*NTU*	number of transfer units
*n*	number of samples
*o*	the output
*ō*	the mean of the output
*p*	pressure, kPa
*R*	correlation coefficient
*T*	temperature, °C
*t*	the target value
*w*	weight
*X*	independent variable
*Y*	dependent variable
**Greeks**	
*p*	density, kg/m^3^
*ε*	moisture exchange effectiveness
*φ*	relative humidity
*ω*	absolute humidity
*σ*	activation function
*z*	weighted sum of the input
**Subscripts**	
a	air
d	dry
i	inlet
j	index, jth data point
m	membrane
min	minimum
max	maximum
o	overall
s	saturation
t	total
tr	transfer
w	wet
